# STOP smoking and alcohol drinking before OPeration for bladder cancer ﻿(the STOP-OP study),﻿ perioperative smoking and alcohol cessation intervention in relation to radical cystectomy: study protocol for a randomised controlled trial

**DOI:** 10.1186/s13063-017-2065-6

**Published:** 2017-07-17

**Authors:** Susanne Vahr Lauridsen, Thordis Thomsen, Peter Thind, Hanne Tønnesen

**Affiliations:** 1 Department of Urology, Copenhagen University Hospital, Rigshospitalet, 2112, Blegdamsvej 9, 2100 Copenhagen, Denmark; 2 Abdominal Centre, Copenhagen University Hospital, Rigshospitalet and University of Copenhagen, Health and Medical Sciences, Copenhagen, Denmark; 3 Clinical Health Promotion Centre, Bispebjerg and Frederiksberg Hospital, Copenhagen University Hospitals, Copenhagen, Denmark; 4 Clinical Health Promotion Centre, Health Sciences, Lund University, Lund, Sweden; 5 Health Science, University of Southern Denmark, Odense, Denmark

**Keywords:** Smoking, Alcohol, Surgery, postoperative complications, Bladder cancer

## Abstract

**Background:**

To evaluate the effect of a smoking-, alcohol- or combined-cessation intervention starting shortly before surgery and lasting 6 weeks on overall complications after radical cystectomy. Secondary objectives are to examine the effect on types and grades of complications, smoking cessation and alcohol cessation, length of hospital stay, health-related quality of life and return to work or habitual level of activity up to 12 months postoperatively.

**Methods/design:**

The study is a multi-institutional randomised clinical trial involving 110 patients with a risky alcohol intake and daily smoking who are scheduled for radical cystectomy. Patients will be randomised to the 6-week Gold Standard Programme (GSP) or treatment as usual (control). The GSP combines patient education and pharmacologic strategies. Smoking and alcohol intake is biochemically validated (blood, urine and breath tests) at the weekly meetings and at follow-up.

**Discussion:**

Herein, we report the design of the STOP-OP study, objectives and accrual up-date. This study will provide new knowledge about how to prevent smoking and alcohol-related postoperative complications at the time of bladder cancer surgery. Till now 77 patients have been enrolled. Patient accrual is expected to be finalised before the end of 2017 and data will be published in 2018.

**Trial registration:**

ClinicalTrials.gov, ID: NCT02188446. Registered on 28 May 2014.

**Electronic supplementary material:**

The online version of this article (doi:10.1186/s13063-017-2065-6) contains supplementary material, which is available to authorized users.

## Background

Postoperative morbidity after radical cystectomy is frequent despite the implementation of fast-track care pathways and robotic-assisted cystectomy [1]. Complication rates vary from 30 to 64% even in high-volume centers [2, 3]. Common complications include infections, ileus and impaired wound healing, all of which are burdensome for the individual patient and costly for society.

Daily smoking and risky alcohol intake (more than two drinks daily) both increase the risk of postoperative morbidity and cancer [4–6]. Smoking is also the major and most modifiable risk factor for development of bladder cancer in both men and women [7].

Intensive smoking- and alcohol-cessation intervention 6–8 weeks before elective surgery reduces the incidence of postoperative morbidity to about half [8–10]. Postoperative smoking-cessation interventions in elective surgery and after fracture surgery might likewise reduce the development of complications [11, 12].

Preoperative intervention lasting 6–8 weeks prior to cancer surgery is, however, unfeasible given the ongoing efforts to accelerate diagnosis and treatment of patients at suspicion of having cancer. In Denmark, the timeframe for intervening before cancer surgery is maximum 14 days, often less, which renders longer-lasting preoperative intervention impossible today. Furthermore, a programme with one preoperative meeting has shown no effect on surgical risk reduction for other groups of cancer patients undergoing surgery [8, 13]. Currently, standard care, therefore, includes written information about the risks of smoking and alcohol in relation to surgery without proactive intervention or systematic referral to cessation services.

Hypothetically, intensive smoking- and alcohol-cessation intervention initiated at the earliest possible time before surgery, and continued for 5 weeks after surgery, could benefit patients undergoing radical cystectomy. Intervention during this period might to some degree improve pathophysiological mechanisms, such as tissue perfusion and oxygen delivery, ciliary and immune function, surgical stress response, arrhythmias and bleeding time [14–17], all of which are beneficial for postoperative recovery. Findings of physiological studies have shown that most of the smoking-induced changes are reversible to some degree, and that the period needed for a substantial improvement is about 6–8 weeks [18, 19]. To our knowledge, the surgical studies on smoking and alcohol intervention for risk reduction have focussed only on either smoking or alcohol cessation in isolation. Since smoking and alcohol drinking often coexist and alcohol use is associated with increased risk of smoking relapse [20, 21], it would be relevant to intervene on both.

The aim of this randomised study is, therefore, to evaluate the effect of a smoking, alcohol or combined cessation intervention starting shortly before surgery, and lasting 6 weeks, on overall complications after radical cystectomy. Secondary objectives are to examine the effect on types and grades of complications, smoking cessation and alcohol cessation, length of hospital stay, health-related quality of life and return to work or habitual level of activity up to 12 months postoperatively.

## Methods/design

**Fig. 1:**
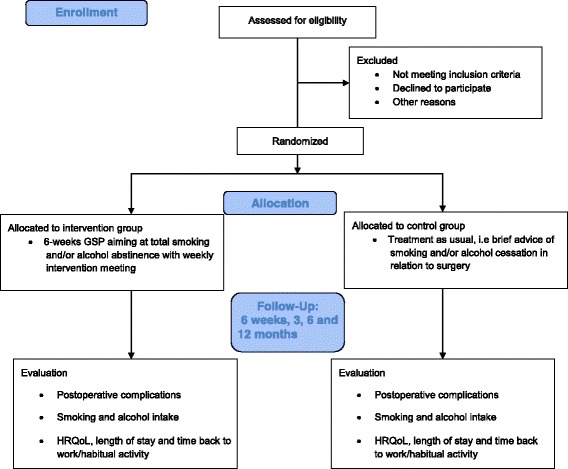
Trial profile

An ongoing multicenter, randomised clinical trial with 1:1 ratio allocation of participants to either: (1) smoking and/or alcohol cessation intervention initiated 1–2 weeks before radical cystectomy and lasting for a total of 6 weeks, or (2) treatment as usual (Fig. 1). The study was initiated in November 2014 and till now 77 patients have been enrolled (Table 1), an average of two to three patients per month. We aim to enrol a total of 110 patients with expected completion of patient accrual in November 2017.

**Table 1:** Accrual of patients to date at the five participating institutions

Institutions	Accrual, *n*
Rigshospitalet, University Hospital of Copenhagen	45
Herlev, University Hospital of Copenhagen	13
Skejby, University Hospital of Aarhus	13
Aalborg University Hospital	5
Odense University Hospital	1

The protocol for this randomised clinical trial is reported in compliance with the Standard Protocol Items: Recommendations for Interventional Trials (SPIRIT) guidelines [22]. See Additional file 1.

### Settings

Currently, all university-affiliated specialised urological centers performing radical cystectomy in Denmark are participating in the trial in order to ensure adequate participant enrolment. Together, they perform approximately 300 radical cystectomies annually.

### Inclusion criteria

Patients scheduled for radical cystectomy for bladder cancer and who smoke daily and/or drink at least 3 units of alcohol daily. One unit contains 12 g ethanol.

### Exclusion criteria

Exclusion criteria are: cognitively unable to provide informed consent; allergy to disulfiram, benzodiazepines or Nicotine Replacement Therapy (NRT); pregnancy and breastfeeding.

## Research procedures

In connection with the initial planning of surgery, the patient’s designated urologist and a trial nurse approach eligible patients with information about the study and the invitation to participate. Trial nurses at each site randomise patients using a computer-generated, stratified, block randomisation scheme [23]. The randomisation system is accessible around the clock via tablets. This ensures immediate randomisation of patients accepting participation and adequate allocation concealment. Block sizes vary from 2 to 8. Stratification takes place for trial site and smoking, risky alcohol drinking or both.

### Intervention

**Fig. 2:**
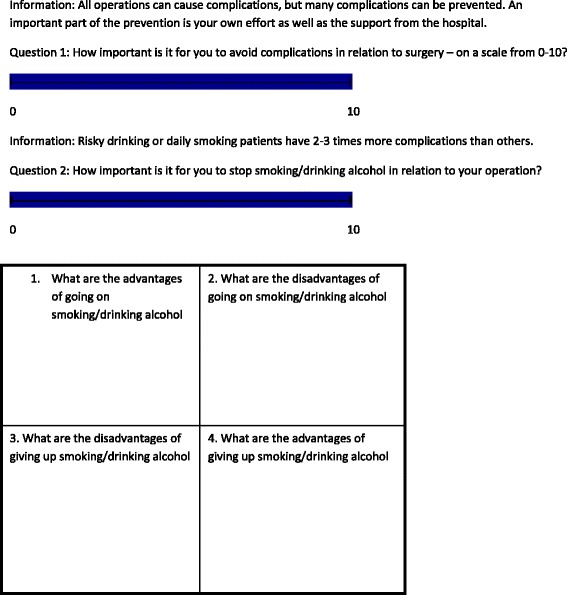
The line for identification and the box for assessing ambivalence

**Fig. 3:**
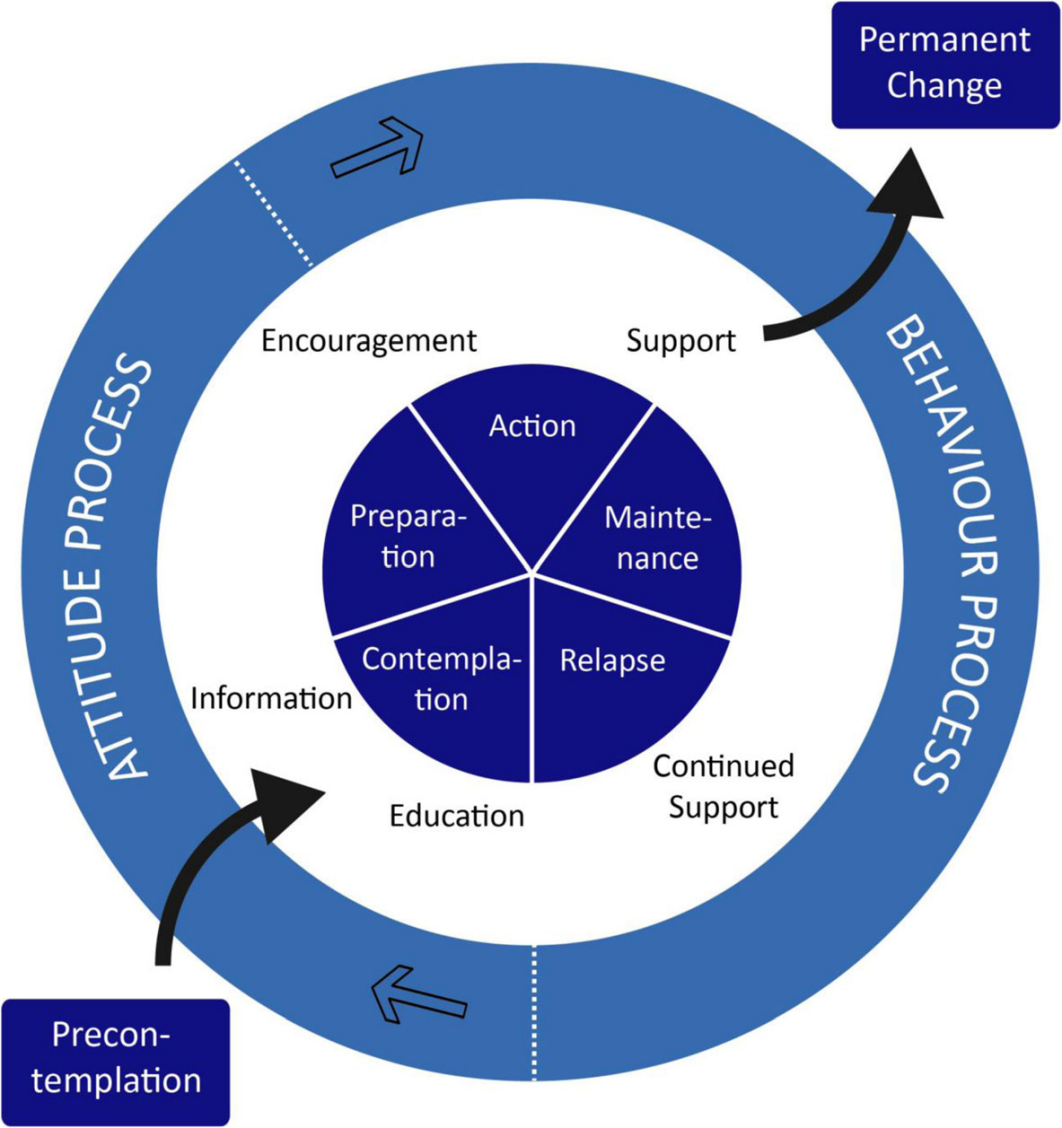
The stages of change model

Patients allocated to the intervention group receive five counselling sessions before and after surgery over 6 weeks with trained smoking- and alcohol-cessation counsellors. Nine registered nurses from the participating sites who have taken part in a 4-day educational programme followed by practical training in the Gold Standard Programme (GSP), provide the intervention (see Table 2) [24]. The project leader (SVL) of the trial ensures that all counsellors follow the principles of the GSP programme by discussion of the intervention regularly. The principles of motivational interviewing, balanced decision-making and the trans-theoretical model of change are the underlying tenets of the programme [25]. These principles describe a general approach to assessing patients’ empowerment using three main tools: the LINE, the BOX and the CIRCLE (see Figs. 2 and 3).

**Table 2:** Gold Standard Programme for smoking and alcohol cessation intervention

Patient education programme First meeting (before admission) ◦ Level of motivation, ambivalence, pros and cons Second meeting (after 1 week) ◦ Dependence, withdrawal symptoms (experience and expectations) Third meeting (after 2 weeks) ◦ Relapse (description and management) Fourth meeting (after 3 weeks) ◦ Benefits of short- and long-term smoking and/or alcohol abstinence Fifth meeting (after 5 weeks) ◦ Continued smoking abstinence and/or reduced alcohol intake following intervention
At each meeting: Smokers: personalised Nicotine Replacement Therapy (NRT) in accordance with patient preferences and nicotine dependency Risky drinkers: thiamine and B vitamins (300 mg × 7 weekly) Alcohol withdrawal prophylaxis and treatment (chlordiazepoxide 10 mg as required) Disulfiram (200 mg × 2 weekly) supervised at weekly meetings (not administrated if patients test positive on breath test) All: haemoglobin, liver enzymes and alcohol biomarkers (blood, urine), carbon monoxide (CO) and alcohol breath test, lung function test
The study medication is provided for free and transportation for the weekly meetings will be reimbursed. Patients have telephone access to the research nurse

Together, the LINE and the BOX facilitate contemplation of change in patients and establish a common ground for supporting change. The CIRCLE is a model representation of the stages that people go through during the process of change. It primarily helps the counsellor to choose how best to support the patient.

At the first meeting the surgical patient is educated about the association between smoking and alcohol intake and the increased risk of postoperative complications. To get the longest period of preoperative quitting the patient is encouraged and supported to quit already at the first meeting. At the second meeting the education focusses on addiction and withdrawal symptoms. Patients may develop stress caused by abstinence from smoking and drinking. In the 6-week intervention period smokers are offered a personalised Nicotine Replacement Therapy (NRT) schedule devised in accordance with patient preferences and nicotine dependency assessed by the Fagerström’s Test for Nicotine Dependency score [26]. Risky drinkers are offered supportive medical treatment against development of mild to moderate withdrawal symptoms (chlordiazepoxide 10 mg, max times 10). In addition, some of the stress is expected to be reduced by the empathic intervention. At the third meeting focus is on relapse management after discharge. At the fourth meeting other benefits of short- and long-term abstinence are on the agenda. At the fifth meeting future lifestyle is discussed together with present experiences and coming challenges and patients are encouraged to continued smoking abstinence and a low alcohol intake within national recommendations.

At each meeting patients are asked if they have experienced side effects of the pharmacological support. Potentially unknown side effects are reported and, if serious, may lead to early termination of the trial.

The study medication is provided for free. All meetings are planned in connection with scheduled visits in the outpatient clinic or at admission to hospital if possible. Patients can contact the research nurse via phone.

### Standard care

**Fig. 4:**
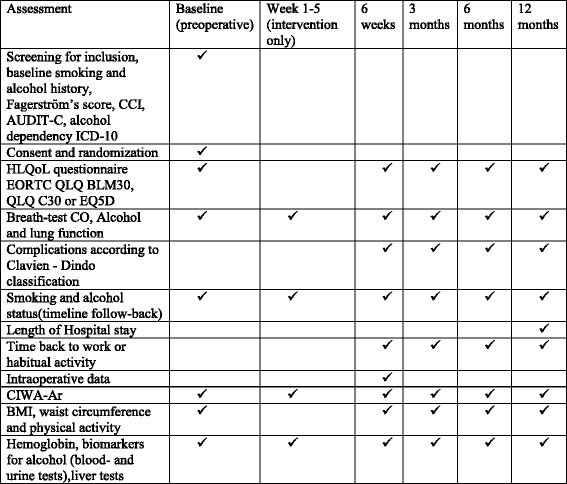
Time schedule for enrolment, interventions and assessments

Apart from information about the background, objectives and potential implications of the current trial, patients allocated to standard care receive the national folders on alcohol and tobacco and surgery. This entails written information about the risks of smoking and drinking in relation to surgery according to national guidelines. Patients are ensured that they are free to access smoking-cessation and/or alcohol-cessation support services in the hospital or elsewhere if they wish. Standard care patients are breath-tested for exhaled carbon monoxide (CO) and alcohol at baseline and follow-up postoperatively (see Fig. 4).

### For both groups

Both groups receive routine procedures regarding general patient information, thromboembolic prophylaxis and antibiotics, anaesthesia, surgical intervention and postoperative care according to the clinical guidelines used at each institution.

In order to find out if patients who decline to participate in the trial differ from those who are enrolled, we ask for permission to follow their perioperative course in the medical journal. Those who accept are asked to provide informed consent.

### Measurements

The primary and secondary outcomes will be evaluated at follow-up points after 6 weeks, 3, 6 and 12 months after surgery (see Fig. 4). All trial data are fed into an electronic Case Report File and will be monitored for completeness. Blood and urine samples are analysed at Labmedicin Skåne, clinical chemistry, Lund, Sweden.

## Objectives of the STOP-OP trial

### Postoperative morbidity

The primary outcome is the proportion of patients who develop any postoperative complication, or death, within 30 days after surgery. Secondary outcomes are: types and grades of complications within 30 and 90 days after surgery assessed using the Clavien-Dindo classification [27] of surgical complications. Evaluation of postoperative complications is performed prospectively through patient interviews at all follow-up meetings and retrospectively by a urologist unaware of the patients’ group allocation.

Previous studies of intensive preoperative smoking cessation interventions report 65% and 49% reductions in overall postoperative complications [10–12], and preoperative alcohol cessation interventions a 74% reduction [9]. We assume that 50% of the project patients develop one or more complications after radical cystectomy. Our aim is to reject or confirm a reduction in the number of patients who develop any postoperative complication from 50% to 25%. With a type-1 error risk of 5% and a type-2 error risk of 20% (80% power), the required sample size is 55 patients in each group.

### Smoking and alcohol cessation

At baseline and at all meetings and follow-up visits, haemoglobin, liver function tests and alcohol biomarkers (carbohydrate-deficient transferrin (CDT) and phosphatidylethanol (PEth)) are determined in both the intervention and control groups. In addition, breath tests measuring alcohol and CO are done. During the intervention, patients report alcohol and/or tobacco use in a log book. Duration of self-reported and validated continuous smoking and/or alcohol abstinence up to 12 months after surgery is reported.

#### Length of hospital stay (LOS)

Length of stay is used as a surrogate for recovery after surgery. We measure number of days from surgery (day 0) till discharge and also number of days in hospital within 90 days after surgery.

### Other outcomes

#### HRQoL

We measure health-related quality of life (HRQoL) outcomes using the generic questionnaire EuroQol-5D (EQ-5D) at baseline and postoperatively at 6 weeks, 3, 6 and 12 months after surgery. In addition, we use the disease-specific European Organisation of Research and Treatment of Cancer Questionnaires EORTC-QLQ-C30 and QLQ-BLM30 for patients with bladder cancer at baseline and postoperatively at 6 and 12 months.

Time to return to work or habitual level of activity is a surrogate for recovery after surgery and will be evaluated within 12 months after surgery.

### Statistics

The data are not expected to be normally distributed, so nonparametric tests are performed. Continuous variables are presented as medians and ranges or means and standard deviations (SD) as appropriate. Categorical variables are presented as counts. Their associated 95% confidence intervals (CI) and *p* values will be provided. The effect of the intervention on risk of postoperative complications and smoking/alcohol abstinence is analysed by Fisher’s exact test. Estimates of the difference between treatment groups will be reported as relative risks (RR) with 95% confidence intervals and *p* values. Effects of the intervention on LOS and HRQoL scores are analysed by Mann-Whitney’s *U* test. Analysis adjusting for institution is also performed by logistic regression models. Effects are considered statistically significant if *p* values are less than 0.05.

We will conduct all analyses using intention-to-treat principles. All analyses are done blinded by an independent researcher. We will use R 3.2.2 [28] to perform all analyses.

## Discussion

The randomised design provides evidence on the highest possible level regarding the effect of an intensive smoking- and/or alcohol-cessation intervention on complications after bladder cancer surgery. Blinding of patients and project staff is not possible or intended as the intervention includes patient education to support abstinence. However, evaluation of postoperative complications is performed blinded by a urology specialist unaware of the patients’ group allocation. Also, the statistical analyses are done blinded by an independent researcher.

Recruitment of patients to the trial is expected to be challenging, as the proportion of bladder cancer patients in Denmark who are current smokers is about 30% [29]. Table 1 shows the difference in recruitment rates among our centres. Previous studies report widely varying participation rates in preoperative smoking cessation interventions ranging from 31 to 96% [8]. The number needed to screen to get one eligible patient to accept participation in alcohol-intervention studies varies from a few up to 70 patients. However, the rates do not differ between RCTs and non-RCTs, or between brief and intensive alcohol-intervention studies [30]. A strength in this study is that we follow common research practice and assess both 7-day-point-prevalence-rates and continuous abstinence during the intervention period validated by biochemical tests. These are regarded as valid, replicable outcome measures and less likely to be biased by faulty recall [31, 32].

This is the first trial to evaluate the effect of both a smoking- and alcohol-cessation intervention on postoperative complications in patients undergoing cancer surgery and thus provides much needed level-1 evidence in this area. From a clinical perspective this may help to improve postoperative outcome and also address the implications of risky drinking and smoking in cancer surgery. From a patient perspective, an optimised perioperative course may minimise any short-term negative impact of surgery on HRQoL. A 6-week period of abstinence from tobacco and alcohol could reduce risky alcohol drinking and motivate long-term smoking cessation and thus benefit psychological HRQoL [33]. From a socioeconomic perspective a potential reduction in postoperative complications would impact the use of health care services and result in cost-savings both in the short and long term. The results from the STOP-OP study will be published in both scientific journals and patient-directed journals.

## Additional file

Additional file 1: SPIRIT 2013 Checklist: recommended items to address in a clinical trial protocol and related documents*. (DOC 121 kb)

## Abbreviations

AUDIT-C: Alcohol Use Disorders Identification Test
CCI: Charlson’s Comorbidity Index
CDT: Carbohydrate-deficient transferrin
CI: Confidence interval
CIWA-Ar: Clinical Institute Withdrawal Assessment-Alcohol, revised
CO: Carbon monoxide
EORTC: European Organisation of Research and Treatment of Cancer
EQ-5D: EuroQol-5D – a standardised instrument for use as a measure of health outcome
GSP: Gold Standard Programme
HRQoL: Health-related quality of life
LOS: Length of stay
NRT: Nicotine Replacement Therapy
PEth: Phosphatidylethanol
QLQ-BLM30: a 30-item questionnaire for patients with muscle invasive bladder cancer
QLQ-C30: A 30-item quality of life questionnaire for cancer patients
RR: Relative risk
SD: Standard deviation
